# Arterial Stiffness as a Predictor of the Index of Atherosclerotic Cardiovascular Disease in Hypertensive Patients

**DOI:** 10.3390/ijerph20042832

**Published:** 2023-02-06

**Authors:** Guili Chang, Yueliang Hu, Qian Ge, Shaoli Chu, Alberto Avolio, Junli Zuo

**Affiliations:** 1 Department of Cardiovascular Medicine, Shanghai Institute of Hypertension, Ruijin Hospital, School of Medicine, Shanghai Jiao Tong University, Shanghai 200025, China; 2 Department of Geriatrics, Ruijin Hospital, School of Medicine, Shanghai Jiao Tong University, Shanghai 200025, China; 3 Macquarie Medical School, Faculty of Medicine, Health and Human Sciences, Macquarie University, Sydney, NSW 2109, Australia

**Keywords:** carotid-femoral pulse velocity, arterial stiffness, atherosclerotic cardiovascular disease, cut-off value, non-invasive hemodynamic indices

## Abstract

Objective: The aim of this study was to evaluate the predictive value of carotid-femoral pulse wave velocity (cfPWV) and cardiovascular disease in the hypertensive population in China and to determine the specific cfPWV cut-off value for assessing future cardiovascular disease (CVD) risk. Methods: This cross-sectional study included 630 hospital patients with primary hypertension and multiple cardiovascular risk factors or complications involving damage to clinical target organs. The study was conducted between July 2007 and October 2008. Atherosclerotic cardiovascular disease (ASCVD) risk calculations were computed according to criteria presented by the American College of Cardiology and the American Heart Association. Patients were stratified by a predefined risk threshold of 10% and divided into two groups: ASCVD ≥ 10% or ASCVD < 10%. cfPWV was used as a marker of arterial stiffness. A receiver operating characteristics (ROC) curve was applied to establish the optimal cfPWV cut-off point to differentiate between participants with and without ASCVD risk. Results: In the study cohort of 630 patients (age 63.55.2 ± 8.6 years, 61.7% male) with primary hypertension, the pressure indices (augmented pressure, augmentation index [AIx], aortic pulse pressure, aortic systolic pressure [SBP]) and Framingham Risk Scores (FRS) were greater in females than in males (*p* < 0.001); ASCVD risk scores and peripheral diastolic pressure (DBP) were higher in males (*p* < 0.05). All hemodynamic indices showed a significant positive correlation with ASCVD risk scores and FRS; AIx was not correlated with ASCVD risk scores. In multivariate logistic analysis, cfPWV was significantly associated with ASCVD risk (OR: 1.324, 95% confidence interval: 1.119–1.565, *p* < 0.001) after adjusting for age, gender, smoking, body mass index, total cholesterol, fasting blood glucose, antihypertensive treatment, statin treatment, and DBP. In the ROC analysis, the area under the curve was 0.758 and 0.672 for cfPWV and aortic SBP (*p* < 0.001 and *p* < 0.001, respectively); the optimal critical value of cfPWV and aortic SBP was 12.45 m/s (sensitivity 63.2%, specificity 77.8%) and 124.5 mmHg (sensitivity 63.9%, specificity 65.3%). Conclusions: cfPWV is significantly correlated with the risk of ASCVD. The best cut-off value of cfPWV for assessing future CVD risk in the hypertensive population in China is 12.45 m/s.

## 1. Introduction

The rapid development of the social economy has had a large impact on the lifestyle of a large proportion of the population in China. Under the influence of factors such as lifestyle changes, urbanization, and population aging, the incidence of cardiovascular disease (CVD) in China is increasing. Currently, it is estimated that 11 million people suffer from coronary heart disease and 4.5 million people have heart failure. There are 245 million patients with hypertension. CVD mortality ranks first among various diseases, and this upward trend is expected to continue in the next decade [1].

In order to reduce the death rate of CVD as much as possible, it is an effective means to actively intervene in high-risk groups (such as patients with hypertension) and improve overall prevention. Arterial stiffness as measured by carotid-femoral pulse wave velocity (cfPWV) is an emerging tool for CVD risk assessment and stratification [2]. In hypertensive patients, elevated cfPWV is associated with an increased risk of cardiovascular death [3]. Previous studies have evaluated atherosclerotic cardiovascular disease (ASCVD) risk in the hypertensive population [4,5].

In the application of cfPWV, the following problems were found: (i) there may be differences in arterial stiffness among different races [6]. Blacks have more common vascular dysfunction than whites; (ii) the cfPWV only reflects the stiffness of the large arteries and cannot fully reflect the stiffness or function of the smaller conduit arteries [7]; (iii) the cfPWV can divide participants into high and low CVD risk groups, but its cut-off value is still not established. A meta-analysis [8] showed that when the cfPWV cut-off value was studied in relation to cardiovascular events and cardiovascular death, the results showed strong heterogeneity. The relationship may be influenced by other confounding factors, including age, race, and gender. Most of the studies involving the cut-off value of cfPWV have been conducted in European and American populations, which may have limitations in their application to the Chinese population.

Therefore, we designed this study to determine whether cfPWV is still an effective predictor of CVD in the hypertensive population in China and to determine the specific cut-off value for assessing future CVD risk. It is hoped that through the findings of this study, high-risk groups will be screened and active interventions put in place to reduce the risk of morbidity and mortality.

## 2. Methods

In this cross-sectional observational study, a total of 630 inpatients (389 males, age 63.26 ± 9.06 years) treated with antihypertensive medication were selected from the Department of Hypertension, Ruijin Hospital, Shanghai, China, between July 2007 and October 2008; all had multiple cardiovascular risk factors or complications involving damage to clinical target organs. The study protocol was approved by the local Ethics Committee. Ethics committee reference number (2017, (1)-1.)

Inclusion criteria included: essential hypertension diagnosed in accordance with guidelines at the time of the study or treatment with antihypertensive medication. Exclusion criteria included any evidence of secondary hypertension, acute cardiovascular and cerebrovascular disease in the past 3 months, any serious illness that threatened life, or any malignant arrhythmia that interfered with the quality of monitoring techniques for arterial stiffness.

After fasting overnight with no caffeinated beverages or smoking 3 h before measurement, all patients underwent blood sampling and standardized questionnaires to collect information on their medical history. Smoking status, medication status, total serum cholesterol, high-density lipoprotein cholesterol, triglyceride, blood glucose, serum creatinine, and urinary ACR were recorded. Current smoking was defined as smoking within one week of measurement of blood pressure and arterial stiffness.

The central aortic pressure waveforms were derived from the radial pressure waveforms with a validated transfer function of the Sphygmocor software, version 8.0 (AtCor Medical, Sydney, Australia). Participants were placed in the supine position with the right upper limb in external rotation. The probe was placed at the strongest point of the right radial artery pulsation. Radial artery pulses were recorded for at least 12 s. The device automatically converted the peripheral pulse wave to a central aortic pressure pulse using a validated transfer function and then generated aortic systolic pressure (SBP) [9], aortic diastolic pressure (DBP), aortic pulse pressure (PP), augmented pressure (AP), and a central augmentation index (Alx). Radial waveforms were calibrated with peripheral SBP (pSBP) and peripheral DBP (pDBP) measured once at the left brachial artery with a validated Omron 705CP oscillometric device (Omron, Kyoto, Japan).

After the office blood pressure measurement, carotid and femoral artery waveforms on the patient’s right side were recorded using a high-fidelity applanation tonometer (Complior SPIV, Artech-Medical, Paris, France). Carotid-femoral pulse velocity (cfPWV) was calculated as the ratio of the direct distance between the measurement points of the carotid and femoral to the pulse transmission time.

ASCVD risk calculations were performed in accordance with the American College of Cardiology (ACC) and the American Heart Association (AHA) recommendations, taking into account age, race, sex, blood pressure, smoking (yes/no), cholesterol, diabetes (yes/no), and status for treatment with antihypertensive medication, statins, and aspirin. After computing the 10-year risk using an ASCVD calculator, patients were stratified by a predefined risk threshold of 10%, and divided into two groups: ASCVD ≥ 10% (Group I) and ASCVD < 10% (Group II).

The Framingham Risk Score (FRS) was calculated using age, sex, diabetes (yes/no), smoking (yes/no), systolic blood pressure, and body mass index (BMI). Absolute CVD percentage over 10 years was classified as low risk (<10%), intermediate risk (10–20%), and high risk (>20%) [10]. This study was approved by the Ethics Committee of Ruijin Hospital, School of Medicine, Shanghai Jiao Tong University. All patients provided written, informed consent.

## 3. Statistical Analysis

All analyses were performed using SPSS 24.0 for Windows (SPSS Inc., Chicago, IL, USA). A two-sided *p* < 0.05 was considered statistically significant. Continuous variables are expressed as mean ± SD. Categorical variables and frequencies were compared with the chi-square (χ^2^) analysis.

Pearson correlation was used to assess the relations between aortic SBP, DBP, AIx, AP, PP, cfPWV, and ASCVD risk scores, or FRS. The association between ASCVD risk scores and cfPWV was assessed by multiple linear regression (adjusting for confounding factors such as age, gender, smoking, BMI, total cholesterol, fasting blood glucose, antihypertensive treatment, statin treatment, and DBP or SBP and aortic SBP or PP and aortic PP).

Backward conditional logistic regression analysis was used to predict ASCVD risk.

A receiver operating characteristics (ROC) curve was applied to establish the optimal cfPWV and aortic SBP cut-off points to differentiate between participants with and without ASCVD risk. An ASCVD threshold of 10% was selected to risk-stratify the patients into either the low-risk (ASCV < 10%) or high-risk (ASCVD ≥ 10%) category. The optimal cut-off point was computed by maximization of Youden’s index (sensitivity + specificity −1) in the ROC curve analysis. A *p*-value of <0.05 was considered statistically significant. An AUC was used as the primary performance evaluation metric for this proposed study. The interpretation of AUC values is such that 0.5 is no discrimination, 0.51–0.69 is a poor test, 0.7–0.79 is acceptable, 0.8–0.89 is excellent, and ≥0.9 is outstanding [11].

## 4. Results

The clinical characteristics of 630 patients are shown in Table 1. The mean age was 63.55 ± 8.60 years, and 389 patients (61.7%) were men. The pressure indices (AP, AIx, aortic PP, aortic SBP) and FRS were greater in females than in males (*p* < 0.001). However, ASCVD and peripheral DBP were higher in males (*p* < 0.05). There were no differences in cfPWV, peripheral SBP, and aortic DBP between males and females.

**Figure 1:**
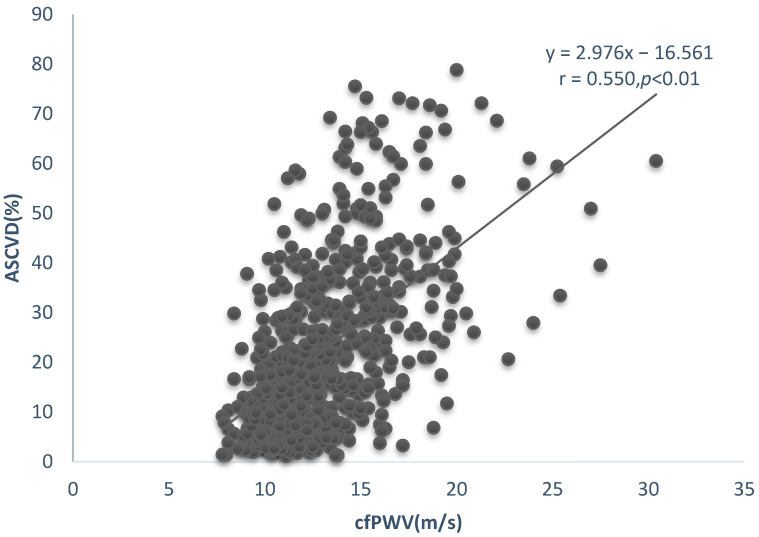
Linear relationship between cfPWV and ASCVD.

**Figure 2:**
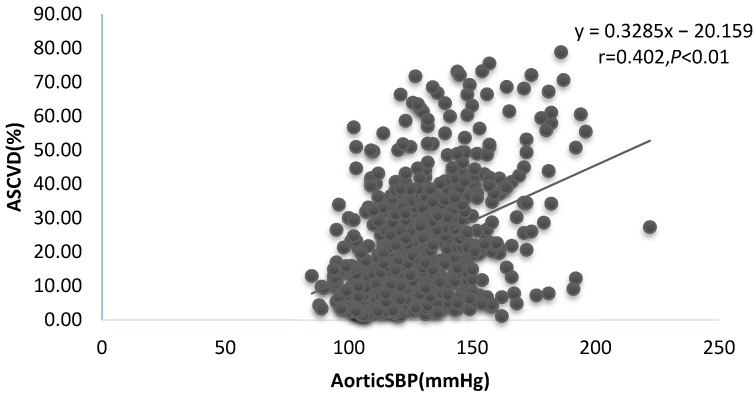
Linear relationship between aortic SBP and ASCVD.

**Figure 3:**
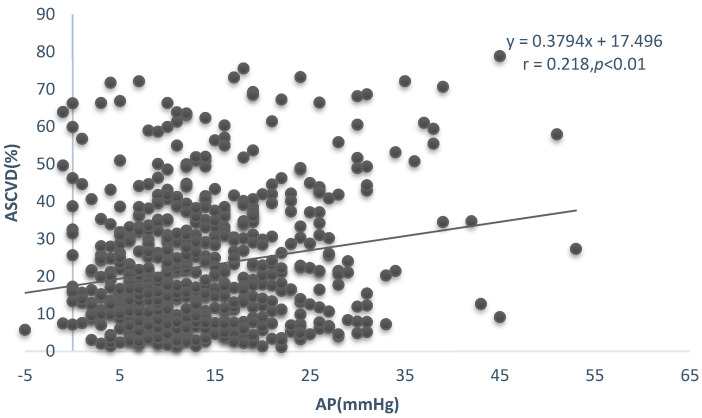
Linear relationship between AP and ASCVD.

Table 2a summarizes the correlations between ASCVD, FRS, and hemodynamic indices. All hemodynamic indices showed a significant positive correlation with ASCVD and FRS, except for Aix, which was not correlated with ASCVD. (Figure 1, Figure 2 and Figure 3). Table 2b summarizes the correlations between cfPWV and age, SBP, TC, HDL, and BMI. It shows a significant positive correlation with age, SBP, and BM except for TC and HDL.

Using ASCVD as the independent continuous variable in multiple linear regression, cfPWV was positively associated with ASCVD (β = 0.173; *p* < 0.001) after adjusting for confounding factors such as age, gender, smoking, BMI, total cholesterol, fasting blood glucose, antihypertensive treatment, statin treatment, eGFR, and DBP (Table 3).

Table 4a and Table 5 show values when we adjusted for age, gender, smoking, BMI, total cholesterol, fasting blood glucose, antihypertensive treatment, statin treatment, eGFR, SBP, aortic SBP, PP, and aortic PP; it was also found that cfPWV was positively associated with ASCVD (β = 0.064; *p* = 0.009 and β = 0.096; *p* < 0.001 respectively).

Table 4b shows values when we adjusted for age, gender, smoking, BMI, total cholesterol, fasting blood glucose, antihypertensive treatment, statin treatment, eGFR, SBP, and aortic SBP; it was found that cfPWV was associated with FRS (β = 0.081; *p* = 0.049).

Patients were divided into two groups: ASCVD ≥ 10% in Group I and ASCVD < 10% in Group II. In the multivariate logistic analysis, cfPWV was significantly associated with ASCVD (OR: 1.324, 95% confidence interval: 1.119–1.565, *p* = 0.001, Table 6), adjusting for age, gender, smoking, BMI, total cholesterol, fasting blood glucose, antihypertensive treatment, statin treatment, and DBP.

**Figure 4:**
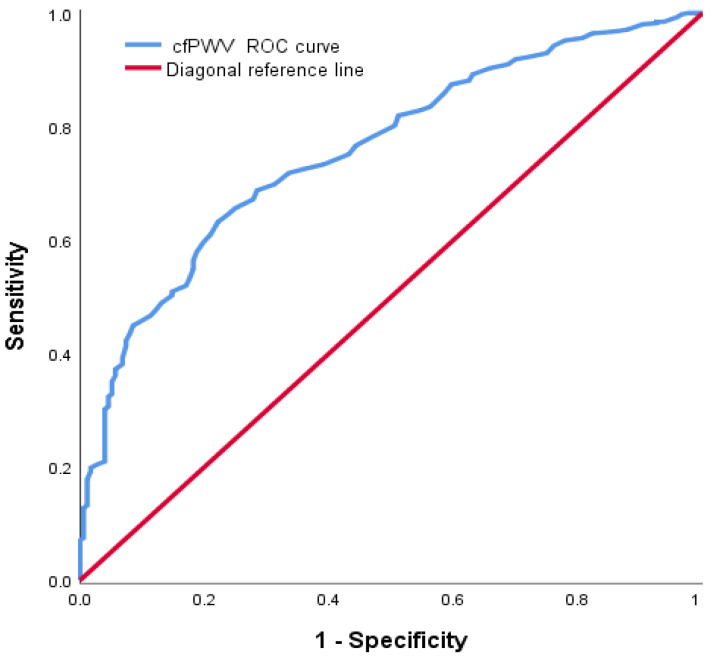
Receiver operating characteristic (ROC) curve to determine the cut-off value of carotid-femoral pulse wave velocity for risk of ASCVD. The cut-off point of 12.45 m/s showed a sensitivity of 63.2%, a specificity of 77.8%, and an area under the curve (AUC) of 0.758.

**Figure 5:**
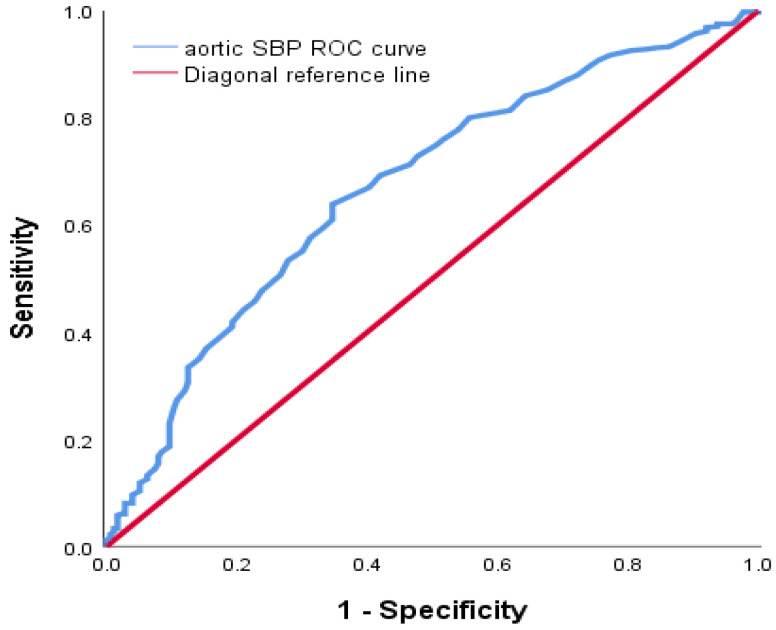
Receiver operating characteristic (ROC) curve to determine the cut-off value of aortic systolic blood pressure for risk of ASCVD. The cut-off point of 124.5 mmHg showed a sensitivity of 63.9%, a specificity of 65.3%, and an area under the curve (AUC) of 0.672.

We performed ROC analysis to obtain the cut-off values of cfPWV and aortic SBP to differentiate between subjects with and without ASCVD risk. However, these associations do not consider covariates in standard ROC curves. The ROC curves showed that 12.45 m/s was the cfPWV cut-off point (AUC: 0.758, sensitivity: 63.2%, specificity: 77.8%) (Figure 4). The ROC curves also showed that 124.5 mmHg was the aortic SBP cut-off point (AUC: 0.672, sensitivity: 63.9%, specificity: 65.3%) (Figure 5).

## 5. Discussion

The most important finding of the study is that after adjusting for age, smoking, gender, total cholesterol, fasting blood glucose, BMI, antihypertensive treatment, statin treatment, and blood pressure, cfPWV is still significantly correlated with the ASCVD risk, with a best cfPWV cut-off value of 12.45 m/s. The increase in arterial stiffness will cause the reflected pressure wave to return to the heart earlier, resulting in increased left ventricular load and potentially decreased pressure during diastole, affecting coronary perfusion thereby increasing the risk of CVD [12].

A study [13] has shown that the measurement method of brachial to ankle PWV (ba-PWV) is simpler and has good repeatability, so it may be a useful arterial stiffness measurement index. ba-PWV has been widely used in East Asian countries (especially Japan) [2]. A study also showed that for dialysis patients, an increased aortic-brachial arterial stiffness gradient (defined as the ratio of cfPWV and carotid-to-radial PWV) was a better predictor of all-cause mortality than cfPWV [14]. However, our study confirmed that after adjusting for many risk factors, cfPWV is still the most reliable measure of arterial stiffness for predicting cardiovascular disease risk, which is consistent with a population study conducted in the community [15]. This may be because the aorta-brachial artery stiffness gradient and ba-PWV are more affected by peripheral arteries. If the stiffness of the aorta increases significantly, but the stiffness of the surrounding arteries only slightly increases or does not increase, the detection effect of the aortic-brachial artery stiffness gradient and ba-PWV will be affected. In the future, this type of study can be repeated in patients with different types of vascular dysfunction to verify the effectiveness of cfPWV. However, hypertensive patients without target organ damage may have a very different profile compared to those that already present with target organ damage, especially coronary artery disease. Thus, in such conditions, cfPWV is not strictly a predictor but can be considered an identifier of target organ damage.

A meta-analysis of most European and American populations [8] showed that the cut-off values of the cfPWV for CVD and CVD death have strong heterogeneity. It is suggested that a cfPWV exceeding 10 m/s should be considered a CVD risk factor [16,17]. There is also a study that defines the cut-off value of high PWV as 90% of an age-specific reference sample [18]. In addition to known determinants such as blood pressure and age, race and gender will all affect the PWV level and cut-off value [13]. For example, a Japanese study suggested that menopause augments the age-related increase in arterial stiffness during the early postmenopausal phase and that this augmentation is probably related, at least in part, to estrogen deficiency [19]. A study conducted on black Americans suggested that nitric oxide-dependent and non-dependent vascular dysfunction may be the cause of ethnic differences in PWV [6]. At present, in addition to our own research, cfPWV is associated with cardiovascular risk factors and may be a risk factor for cardiovascular events; however the cfPWV cut-off data for the Chinese population are not yet sufficient and need to be further improved by large-scale studies. Current data suggest that individuals with a cfPWV > 12.45 m/s should be alert to the risk of future CVD and require active intervention.

In general characteristics of the study population, we found that the pressure indices and FRS were greater in females, but those for ASCVD were higher in males with slightly high cfPWV, which might be caused by unhealthy lifestyles such as a greater prevalence of smoking in more than 50% of males and more antihypertensive medication leading to lower blood pressure in males compared to females. The gender difference in cfPWV as a marker to predict ASCVD would need to be studied in the future.

Our study also considered the correlation between cfPWV, BP, and target organ damage (TOD) such as IMT and eGFR and the risk of CVD. Previous studies have demonstrated a significant association between carotid IMT (cIMT) and ASCVD risk [20,21]. In addition, a study has also confirmed that eGFR is significantly correlated with ASCVD risk [22]. Another study has demonstrated that cIMT is significantly associated with ASCVD risk, while eGFR was not found to be predictive [5]. In our study, we have shown a significant correlation of both cIMT and eGFR with ASCVD risk. Despite the effect of eGFR on subclinical target organ damage, there was no significant association between eGFR and ASCVD in this study after adjusting for multiple risk factors.

In addition, the relationship with ASCVD is stronger than that of FRS. So, the 2019ACC/AHA Guidelines suggest that adults being evaluated for CVD prevention undergo ASCVD risk estimation before starting on antihypertensive or statin therapy [23]. Adjusting for risk factors, including blood pressure, it was found that cfPWV was marginally associated with FRS. Therefore, cfPWV can predict the FRS dependency of blood pressure, whereas cfPWV, when corrected for blood pressure, could better predict the association with ASCVD.

The results suggested that both peripheral and central blood pressure are significantly correlated with ASCVD. The predictive value of peripheral systolic blood pressure for ASCVD is higher than that of central systolic blood pressure. This is at variance with other studies showing that central aortic blood pressure has a stronger correlation with the predictability of related ASCVD and left ventricular hypertrophy [24]. Peripheral blood pressure is associated with central aortic blood pressure and arterial stiffness, which can only reflect heart function to a limited extent [25]. Previous studies have suggested that in the general Chinese community, central systolic blood pressure independently predicts the mortality of cardiovascular diseases [26]. Our previous study [27] also suggested that central blood pressure is better than peripheral blood pressure at predicting CVD events. One Australian study [28] suggested that brachial artery blood pressure rather than non-invasive measurement of central pressure can predict the prognosis of elderly women with hypertension.

The inconsistent conclusions of the above research may have the following implications: (i) Some antihypertensive drugs may have different effects on central and peripheral blood pressure, which are often not considered in the analysis. The ASCOT trial [29] suggested that calcium antagonists ± angiotensin inhibitors can reduce the risk of cardiovascular events compared to β-receptor blockers ± diuretics, and although antihypertensive therapy has similar effects on brachial blood pressure, the calcium antagonist ± angiotensin inhibitor regimen has a more obvious effect on the reduction of central blood pressure. It is speculated that the decrease in the incidence of CVD events in patients is due to the greater reduction in central blood pressure. (ii) What we use is the central blood pressure calculated by a mathematical model, which may have different variability distributions in different cohorts in relation to the accuracy of measurement of cuff sphygmomanometric pressure. (iii) Statins may influence the stiffness of peripheral arteries [30]. In areas with relatively developed medical treatment (such as Shanghai), the proportion of statins taken is very high, which could affect the results of studies conducted in the area.

In addition, our study also suggested that the optimal cut-off value for aortic systolic pressure is 124.5 mmHg. In recent years, many studies have discussed the optimal blood pressure threshold. The SPRINT study pointed out that a peripheral systolic blood pressure drop below 121.4 mmHg can reduce the risk of further cardiovascular events in non-diabetic patients [31]. The Blood Pressure Lowering Treatment Trialists Collaboration (BPLTTC) research published at ESC 2020 suggested that every 5 mmHg reduction in SBP can reduce the relative risk of major cardiovascular events by approximately 10%. The participants’ blood pressure levels at the time of enrollment did not change the cardiovascular benefits brought by antihypertensive therapy. Another new study [32] also suggested that starting from peripheral systolic blood pressure levels as low as 90 mmHg, the presence of calcium in the coronary arteries seems to gradually increase. As the systolic blood pressure level increases, the risk of ASCVD also increases. These findings support the theory that the lower the blood pressure, the better the prognosis. The noninvasive measurement of central aortic pressure can account for the difference between central and peripheral systolic pressure that can be of the order of 10 to 14 mmHg [33]. Since the degree of blood pressure amplification is affected by arterial stiffness, the relevant central arterial pressure threshold needs to be further investigated. However, these associations are only significant in a hypertensive population that is currently receiving treatment. Thus, these findings cannot be directly compared to those in other general population-based studies.

## 6. Conclusions

The findings of this study show that cfPWV is significantly correlated with ASCVD risk. The best cut-off value of cfPWV for assessing future CVD risk in the hypertensive population in China is 12.45 m/s.

## 7. Limitations

Our research has the following limitations: (i) The study population cannot represent the entire Chinese population, as the study was conducted on local hypertensive patients in Shanghai. After obtaining preliminary results, it is necessary to subdivide groups of different genders and ages. (ii) As a cross-sectional study, only the Framingham score is used to predict the risk of CVD, and long-term follow-up is necessary in the future. (iii) Although accounting for as many risk factors as possible, the influence of certain factors was not considered, such as baseline antihypertensive drugs and the cfPWV value. All the cfPWV measures were conducted in the morning, as stress-conditions (reflected by cortisol) greatly affect vascular compliance. However, the effect of cortisol was not taken into account. Finally, inter and intra-variability of PWV measures remain a methodological limitation in our study.

## Figures and Tables

**Table 1:** General characteristics of the study population.

	Total *n* = 630	Male *n* = 389	Female *n* = 241	*p*
Age (Y)	63.55 ± 8.60	63.26 ± 9.06	64.02 ± 7.8	0.285
SBP (mmHg)	143.71 ± 21.76	142.4 ± 22.04	145.81 ± 21.19	0.056
DBP (mmHg)	77.75 ± 10.83	78.58 ± 11.32	76.41 ± 9.87	0.034
HR (bpm)	69.75 ± 10.67	69.65 ± 11.09	69.92 ± 9.96	0.758
BMI (kg/m^2^)	26.05 ± 3.20	26.19 ± 3.03	25.82 ± 3.46	0.166
Cr (μmol/L)	81.70 ± 27.81	91.31 ± 28.04	66.25 ± 19.17	0.000
FBG (mmol/L)	5.99 ± 2.07	5.97 ± 2.23	6.03 ± 1.78	0.703
TG (mmol/L)	2.00 ± 1.29	2.01 ± 1.31	1.98 ± 1.27	0.755
TC (mmol/L)	4.90 ± 1.04	4.68 ± 1.01	5.26 ± 0.98	0.000
LDL-c (mmol/L)	2.93 ± 0.88	2.82 ± 0.85	3.11 ± 0.88	0.000
HDL-c (mmol/L)	1.28 ± 0.37	1.18 ± 0.32	1.43 ± 0.39	0.000
Aortic SBP (mmHg)	129.66 ± 20.47	127.2 ± 20.39	133.53 ± 20.03	0.000
Aortic DBP (mmHg)	78.77 ± 10.65	79.36 ± 11.04	77.84 ± 9.96	0.084
Aortic PP (mmHg)	50.92 ± 15.80	47.93 ± 15.11	55.66 ± 15.75	0.000
AP (mmHg)	13.74 ± 8.41	11.56 ± 7.5	17.2 ± 8.63	0.000
AIX (%)	25.61 ± 10.42	22.71 ± 9.71	30.21 ± 9.85	0.000
cfPWV (m/s)	13.17 ± 3.11	13.29 ± 3.16	12.99 ± 3.02	0.233
eGFR (ml/min)	85.18 ± 21.49	83.29 ± 21.46	88.21 ± 21.22	0.005
cIMT (mm)	0.78 ± 0.18	0.79 ± 0.19	0.77 ± 0.16	0.163
Smoker (n/%)	225 (31.7%)	221 (56.8%)	4 (1.7%)	0.000
ASCVD (%)	22.64 ± 16.83	26.47 ± 16.31	16.47 ± 15.83	0.000
FRS (%)	5.23 ± 5.56	3.93 ± 4.73	7.33 ± 6.15	0.000
DM (n/%)	231 (36.7%)	139 (35.7%)	92 (38.2%)	0.537
Statin treatment (n/%)	195 (31.0%)	124 (31.9%)	71 (29.5%)	0.524
target level of LDL-c (n/%)	228 (36.2%)	165 (42.4%)	63 (26.1%)	0.000
Hypertension duration (Y)	19.89 ± 12.72	19.69 ± 12.76	20.20 ± 12.71	0.644
Hypertension grading				
Grade 1	344 (54.6%)	213 (54.8%)	131 (54.4%)	0.624
Grade 2	173 (27.5%)	104 (26.7%)	69 (28.6%)	
Grade 3	113 (17.9%)	72 (18.5%)	41 (17.0%)	
Target blood pressure	290 (46%)	190 (48.8%)	100 (41.5%)	0.072
Antihypertensive therapy				
CCB	446 (70.8%)	271 (69.7%)	175 (72.6%)	0.429
ACEI	116 (18.4%)	87 (22.4%)	29 (12.0%)	0.001
ARB	323 (51.3%)	195 (50.1%)	128 (53.1%)	0.467
Beta blocker	212 (33.7%)	149 (38.3%)	63 (26.1%)	0.002
Diuretic	113 (17.9%)	63 (16.2%)	50 (20.7%)	0.148

Data are mean ± SD or percentage as marked. *p*-value: independent *t*-test analysis of variance for numeric variables and chi-square test for categorical variables. Abbreviations: BMI: body mass index; SBP: systolic blood pressure; DBP: diastolic blood pressure; FBG: fasting plasma glucose; LDL: low density lipoprotein, HDL: high density lipoprotein; TG: triglycerides, TC: total cholesterol; eGFR: estimated glomerular filtration rate; cIMT: carotid intima-media thickness; cfPWV: carotid-femoral pulse wave velocity; AIX: augmentation index; AP: augmentation pressure; PP: pulse pressure; HR: heart rate; ASCVD: 10-year Kaplan-Meier ASCVD risk rate; FRS: Framingham risk score; CCB: calcium channel blocker; ACEI: angiotensin converting enzyme inhibitor; ARB: angiotensin II receptor blocker.

**Table 2:** (a) Pearson correlation coefficients among variables; (b) Pearson correlation coefficients between cfPWV correlate with variables.

**(a) Pearson correlation coefficients among variables**				
	**ASCVD (%)**		**FRS(%)**	
	**Pearson *r***	***p***	**Pearson *r***	***p***
cfPWV (m/s)	0.550 **	0.000	0.327 **	0.000
AorticSBP (mmHg)	0.402 **	0.000	0.351 **	0.000
AorticDBP (mmHg)	0.103 *	0.010	0.113 **	0.005
Aortic PP (mmHg)	0.449 **	0.000	0.375 **	0.000
AIX (%)	−0.050	0.217	0.169 **	0.000
AP (mmHg)	0.218 **	0.000	0.312 **	0.000
DBP (mmHg)	0.098 *	0.014	0.092 *	0.021
PP (mmHg)	0.526 **	0.000	0.365 **	0.000
eGFR (ml/min)	−0.289 **	0.000	−0.164 **	0.000
cIMT (mm)	0.269 **	0.000	0.149 **	0.000
**(b) Pearson correlation coefficients between cfPWV correlate with variables**				
**cf PWV (m/s)**				
	***p***		**r**	
Age (Y)	0.464 **		0.000	
SBP (mmHg)	0.501 **		0.000	
TC (mmol/L)	−0.022		0.579	
HDL (mmol/L)	−0.005		0.898	
BMI (kg/m^2^)	−0.086 *		0.031	

* *p* < 0.05; ** *p* < 0.01; Abbreviations: SBP: systolic blood pressure; DBP: diastolic blood pressure; cfPWV: carotid-femoral pulse wave velocity; AIX: augmentation index; AP: augmentation pressure; PP: pulse pressure; eGFR: estimated glomerular filtration rate; cIMT: carotid intima-media thickness.

**Table 3:** Linear regression analysis of associations between ASCVD and cfPWV.

	B	SE	Beta	t	*p*
(Constant)	−103.607	4.988		−20.772	0.000
Age (Y)	1.397	0.051	0.716	27.350	0.000
smoking	7.149	0.928	0.204	7.708	0.000
DBP (mmHg)	0.239	0.035	0.154	6.793	0.000
cfPWV (m/s)	0.930	0.136	0.173	6.828	0.000
gender	−7.114	0.915	−0.206	−7.776	0.000
FBG (mmol/L)	1.165	0.174	0.143	6.703	0.000
TC (mmol/L)	1.403	0.356	0.087	3.935	0.000

Abbreviations: FBG: fasting plasma glucose; TC: total cholesterol; cfPWV: carotid-femoral pulse wave velocity; DBP: diastolic blood pressure; Adjusted for age, gender, smoking, and body mass index, total cholesterol, fasting blood glucose, antihypertensive treatment, statin treatment, eGFR, and DBP.

**Table 4:** Linear regression analysis of associations between cardiovascular risk scores and cfPWV.

**(a) Linear regression analysis of associations between ASCVD and cfPWV, adjusting for SBP and aortic SBP**					
	**B**	**SE**	**Beta**	**t**	***p***
(Constant)	−104.405	3.763		−27.743	0.000
Age (Y)	1.301	0.044	0.669	29.825	0.000
SBP (mmHg)	0.246	0.017	0.319	14.389	0.000
gender	−8.727	0.825	−0.255	−10.581	0.000
Smoking	6.996	0.837	0.201	8.359	0.000
FBG (mmol/L)	1.066	0.156	0.132	6.821	0.000
TC (mmol/L)	1.536	0.32	0.096	4.806	0.000
cfPWV (m/s)	0.345	0.132	0.064	2.62	0.009
**(b) Linear regression analysis of associations between FRS and cfPWV, adjusting for SBP and aortic SBP.**					
	**B**	**SE**	**Beta**	**t**	***p***
(Constant)	−26.669	2.428		−10.985	0.000
Age (Y)	0.243	0.026	0.374	9.530	0.000
gender	4.441	0.470	0.388	9.441	0.000
SBP (mmHg)	0.050	0.010	0.195	5.161	0.000
smoking	2.765	0.474	0.237	5.838	0.000
Statin therapy	−1.187	0.402	−0.098	−2.951	0.003
TC (mmol/L)	0.453	0.184	0.085	2.467	0.014
eGFR (ml/min)	−0.020	0.009	−0.076	−2.235	0.026
cfPWV (m/s)	0.146	0.074	0.081	1.976	0.049

Abbreviations: FBG: fasting plasma glucose; TC: total cholesterol; eGFR: estimated glomerular filtration rate; cfPWV: carotid-femoral pulse wave velocity; SBP: systolic blood pressure; ASCVD: 10-year Kaplan-Meier ASCVD risk rate. Adjusting for age, gender, smoking, and BMI, total cholesterol, fasting blood glucose, eGFR, antihypertensive treatment, statin treatment, SBP, and aortic SBP. FRS: Framingham risk score. Adjusted for age, gender, smoking, and body mass index, total cholesterol, and fasting blood glucose.

**Table 5:** Linear regression analysis of associations between ASCVD and cfPWV adjusting for PP and aortic PP.

	B	SE	Beta	t	*p*
(Constant)	−81.339	3.485		−23.340	0.000
Age (Y)	1.181	0.046	0.607	25.758	0.000
smoking	6.777	0.858	0.194	7.903	0.000
PP (mmHg)	0.289	0.022	0.304	12.921	0.000
gender	−9.431	0.853	−0.275	−11.055	0.000
FBG (mmol/L)	1.120	0.159	0.139	7.024	0.000
TC (mmol/L)	1.510	0.332	0.094	4.545	0.000
cfPWV (m/s)	0.515	0.131	0.096	3.936	0.000
statin treatment	−1.529	0.722	−0.042	−2.118	0.035

Abbreviations: FBG: fasting plasma glucose; TC: total cholesterol; cfPWV: carotid-femoral pulse wave velocity; PP: pulse pressure; Adjusted for age, gender, smoking, and BMI, total cholesterol, fasting blood glucose, eGFR, antihypertensive treatment, statin treatment, PP, and aortic PP.

**Table 6:** Logistic regression analysis between several atherosclerosis risk factors and ASCVD.

	B	SE	Wald	*p*	OR (95%CI)
Age (Y)	0.455	0.049	84.832	0.000	1.576 (1.430~1.736)
smoking	4.047	0.597	46.039	0.000	57.248 (17.784~184.29)
gender	−2.777	0.449	38.317	0.000	0.062 (0.026~0.150)
BMI (kg/m^2^)	0.148	0.056	6.958	0.008	1.159 (1.039~1.294)
FBG (mmol/L)	0.669	0.157	18.141	0.000	1.953 (1.435~2.658)
TC (mmol/L)	0.298	0.174	2.915	0.088	1.347 (0.957~1.895)
DBP (mmHg)	0.111	0.022	26.102	0.000	1.117 (1.071~1.165)
cfPWV (m/s)	0.280	0.086	10.747	0.001	1.324 (1.119~1.565)
Statin treatment	0.196	0.379	0.266	0.606	1.216 (0.578~2.556)
antihypertensive treatnt	0.202	0.688	0.086	0.769	1.224 (0.318~4.712)
(Constant)	−44.852	4.902	83.734	0.000	0.000

Abbreviations: BMI: body mass index; FBG: fasting plasma glucose; TC: total cholesterol; cfPWV: carotid-femoral pulse wave velocity; DBP: diastolic blood pressure; Adjusted for age, gender, smoking, and BMI, total cholesterol, fasting blood glucose, antihypertensive treatment, statin treatment, and DBP.

## Data Availability

The data presented in this study are available on request from the corresponding author J.Z. (zjl12616@rjh.com.cn). The data are not publicly available due to patient privacy.
